# Chloroquine resistance before and after its withdrawal in Kenya

**DOI:** 10.1186/1475-2875-8-106

**Published:** 2009-05-18

**Authors:** Leah Mwai, Edwin Ochong, Abdi Abdirahman, Steven M Kiara, Steve Ward, Gilbert Kokwaro, Philip Sasi, Kevin Marsh, Steffen Borrmann, Margaret Mackinnon, Alexis Nzila

**Affiliations:** 1 Kenya Medical Research Institute (KEMRI)/Wellcome Trust Collaborative Research Programme, PO Box 230, 80108, Kilifi, Kenya; 2 University of Oxford, Nuffield Department of Medicine, John Radcliffe Hospital, Oxford, UK; 3 Liverpool School of Tropical Medicine, Pembroke Place, Liverpool, L3 5QA, UK; 4 Kenya Medical Research Institute (KEMRI)/Wellcome Trust Collaborative Research Programme, PO Box 43640, 00100, Nairobi, Kenya; 5 Heidelberg University School of Medicine, Institute of Hygiene, FRG, Heidelberg, Germany; 6 Department of Pathology, University of Cambridge, Tennis Court Rd, Cambridge, CB2 1QP, UK; 7 Department of Pharmacology and Therapeutics, University of Liverpool, Liverpool, UK

## Abstract

**Background:**

The spread of resistance to chloroquine (CQ) led to its withdrawal from use in most countries in sub-Saharan Africa in the 1990s. In Malawi, this withdrawal was followed by a rapid reduction in the frequency of resistance to the point where the drug is now considered to be effective once again, just nine years after its withdrawal. In this report, the polymorphisms of markers associated with CQ-resistance against *Plasmodium falciparum *isolates from coastal Kenya (Kilifi) were investigated, from 1993, prior to the withdrawal of CQ, to 2006, seven years after its withdrawal. Changes to those that occurred in the dihydrofolate reductase gene (*dhfr*) that confers resistance to the replacement drug, pyrimethamine/sulphadoxine were also compared.

**Methods:**

Mutations associated with CQ resistance, at codons 76 of *pfcrt*, at 86 of *pfmdr1*, and at codons 51, 59 and 164 of *dhfr *were analysed using PCR-restriction enzyme methods. In total, 406, 240 and 323 isolates were genotyped for *pfcrt*-76, *pfmdr1*-86 and *dhfr*, respectively.

**Results:**

From 1993 to 2006, the frequency of the *pfcrt*-76 mutant significantly decreased from around 95% to 60%, while the frequency of *pfmdr1*-86 did not decline, remaining around 75%. Though the frequency of *dhfr *mutants was already high (around 80%) at the start of the study, this frequency increased to above 95% during the study period. Mutation at codon 164 of *dhf*r was analysed in 2006 samples, and none of them had this mutation.

**Conclusion:**

In accord with the study in Malawi, a reduction in resistance to CQ following official withdrawal in 1999 was found, but unlike Malawi, the decline of resistance to CQ in Kilifi was much slower. It is estimated that, at current rates of decline, it will take 13 more years for the clinical efficacy of CQ to be restored in Kilifi. In addition, CQ resistance was declining before the drug's official withdrawal, suggesting that, prior to the official ban, the use of CQ had decreased, probably due to its poor clinical effectiveness.

## Background

From the 1940s up to the 1990s, chloroquine (CQ) was the mainstay of malaria therapy worldwide. The selection of *Plasmodium falciparum*-resistant isolates was first reported in South-East Asia and South America in the 1950s [1] and by the 1970s, CQ was no longer effective in these parts of the world. In Africa, CQ-resistant isolates only emerged in the 1970s. However, within 10 years, the level of resistance to CQ had risen rapidly [1], both in southern and eastern Africa [2]. In 1993, Malawi was the first African country to replace CQ as the first-line treatment of uncomplicated malaria with the antifolate combination sulphadoxine/pyrimethamine (SP) [3,4]. In 1999, Kenya also replaced CQ with SP [5]. Other countries, such as Uganda and Tanzania followed suit soon after [6,7].

Recent studies in Malawi have indicated that CQ resistance may revert to sensitivity within a decade of withdrawal of the drug, based on analysis of the population frequencies of the mutations known to cause or be associated with CQ resistance, *pfcrt *and *pfmdr1 *genes [3], and assessment of *in vitro *activity and *in vivo *efficacy of the drug [8,9]. This reversal indicates that the *pfcrt *mutants may be loaded with a substantial fitness cost in the absence of drugs, thus leading to their decline in frequency once drug pressure is removed. This raises the possibility of re-introducing this safe and affordable drug for treatment of malaria in Africa [4]. In this paper, are reported the analyses of markers associated with CQ resistance (*pfcrt *and *pfmdr1*) and the *in vitro *activity of CQ against *P. falciparum *isolates collected in an area of high transmission in coastal Kenya (Kilifi), from 1993, prior to the removal of CQ, to 2006, seven years after its withdrawal. In the same area, these changes that occurred in the dihydrofolate reductase gene (*dhfr*), which confers resistance to the replacement drug SP were also compared [10,11].

## Methods

### Samples

A retrospective analasysis was carried out on blood samples collected from patients during several clinical trials of anti-malarial drugs conducted in Kilifi from 1993 to 2006. The 1993–2003 samples originated from several clinical trials of SP, chlorproguanil/dapsone (Lapdap™), and SP-artesunate [12-15] and were collected before drug treatment. Others were collected in 2006 from infected patients both before and after treatment in clinical trials of artemether/lumefantrine (AL)(Coartem™) versus piperaquine-dihydroartemisinin (Artekin^®^) [unpublished data], and amodiaquine (AQ) [16]. No samples were collected in 1996, 2004 and 2005. Blood samples (~50 μl) were spotted onto filter paper, air-dried and stored in plastic bags with silica gel at ambient temperature. Around thirty samples were selected per year and analysed for *P. falciparum *mutation at codon 76 (Lys to Thr) of *pfcrt (pfcrt-76) *and at codon 86 (Asn to Tyr) *of pfmdr1 (pfmdr1–86)*, and at codons 51,59 and 164 of *dhfr*.

### Parasite adaptation and drug assays

A subset of the 2006 samples were adapted *in vitro *for long-term culture and their chemosensitivity profiles to CQ were analysed. Briefly, parasite adaptation was carried out as follows: 1 ml of patient venous blood was collected and added to 5 ml of transport medium (RPMI-1640 containing 10% albumin). Parasites in this medium could be kept at 4°C for 4 days before the *in vitro *adaptation or cryopreserved in liquid nitrogen and adapted later. Fresh parasites were put in culture after washing the suspension with normal RPMI-1640 (centrifugation at 2500 *g *for 15 min); cryopreserved parasites were thawed, washed and put into *in vitro *culture according to standard protocols of cryopreservation [16]. Parasitaemias were monitored until a per cycle increase in parasitaemia greater than two-fold was reached, at which point the culture was diluted by a factor >2 and if parasitaemia increased again (greater than two-fold), the culture was then considered "adapted": this process lasted two to eight weeks. Once parasites were adapted, their chemosensitivity to CQ was determined by the radioisotope incorporation method [17]. Results were expressed as the drug concentration required for 50% inhibition of [^3^H] hypoxanthine incorporation into parasite nucleic acid (IC_50_), using regression analysis of the dose-response curve. Fifty microlitres of culture from these adapted parasites was spotted onto filter paper for DNA extraction and genotyping.

### Genotyping

Parasite genomic material from filter paper was prepared using the methanol procedure described elsewhere [18]. The detection of single-base changes at *pfcrt-76 *and *pfmdr1–86 *was carried out using the PCR-restriction enzyme protocol described elsewhere [3]. Point mutations in codons 51, 59 and 164 of *dhfr *were analysed as described elsewhere [18,19]. The analysis of mutations at codon 164 of *dhfr *of samples was not carried out routinely, since previously reports showed that this mutation was not present at detectable levels in our study site and other African sites where SP resistance is high [20,21]. However, in light of reports on the emergence of 164 *dhfr *mutant in Africa [22-24], the 164 codon of *dhfr *in isolates collected in 2006 was analysed. Mutation at codon 108 was not analysed since the presence of a mutation at codon 51 and/or 59 indicates that the parasite carries the resistant mutation at codon 108 [10,11].

### Statistical analyses

Frequencies of mutations at *pfcrt-76*, *pfmdr1–86 *and *dhfr *(codons 51 and 59) alleles were calculated as the proportion of samples carrying the mutant form out of the total of all samples carrying either only the mutant form or only the wild-type form: thus samples carrying both wild-type and mutant forms, in which relative frequencies could not be determined, were excluded from the denominator and numerator. In the case of *dhfr*, those samples with either a mutation at *dhfr-51 *(from the amino acid Asn to Ile) or *dhfr-59 *(from Cys to Arg) were denoted as 'double mutants'. Those with mutations at both *dhfr-51 *and *dhfr-59 *were denoted as 'triple mutants' and those with or without a resistant allele mutation at *dhfr-*108 (Ser to Asn) were pooled and denoted as wild-type/single mutants ('WT/single mutants'). The data were first analysed by treating these as three haplotypes, and then by treating the double mutant forms as two separate alleles to give four haplotypes.

As some of the samples were collected from patients enrolled in clinical trials, an analysis was performed to determine the potential bias to population frequencies caused by drug treatment of the patients from which the parasites were sampled. This was done by comparing the frequencies of each mutant before versus after drug treatment using a Fisher's Exact test for each trial separately. Thereafter, an analysis was carried out for trends in the frequencies of mutants through time with and then without the post-treatment samples included in the data, by fitting a logistic regression model to the frequency data with generation as a linear covariate and assuming three parasite generations per year (an average of previously used estimates [25,26] and consistent with estimates of durations of infection in children [27]). The selection coefficient, *s*, which describes how much more (or less) fit the resistant mutant is relative to the wild-type, and is a function of the amount of drug pressure and also the cost of resistance in the absence of drug pressure [28], was calculated from the estimated slope of the logistic regression curve fitted to the observed allele frequencies over the generations, viz:

(1)

where the slope of this line is [29], *w*_R _and *w*_S _are the fitnesses of the resistant and wild-type alleles, respectively, 'log' denotes the natural logarithm, *p*_R _and *p*_S _are the relative population frequencies of the resistant and wild-type allele with *p*_R _= 1-*p*_S_, and the superscripts denote the generation number. Solving for *s *is straightforward in the case of two alleles, but in the case of more alleles, as for *dhfr*, this equation cannot be directly used to estimate the selection coefficient because it gives the fitness of one allele relative to all other alleles combined. In this case, the expected slope for allele *i *(*slope*_*i*_) becomes  giving rise to allelic fitnesses of:

(2)

with *i *= 1,2,3 for single/WT, double and triple mutants, respectively, and where *j *denotes all alleles other than *i*. After setting *w*_1 _= 1, Equation (2) can be solved for *w*_2 _and *w*_3 _by substituting in the observed values for *p*_*i *_and *p*_*j *_and minimizing the sum of squared differences between the observed *slope*_*i*_'s (i.e., those obtained by logistic regression) and their expected values, (i.e., those in Equation (2)). Note, however, that *w*_*i *_now depends on the frequencies of the other alleles: since these change with time, Equation (2) is an approximation [30]. In the case of *dhfr *in this study, the single and double mutants changed relatively little with time and so the approximation is likely to be accurate.

Confidence intervals (95%) on estimates of *s *were calculated by taking the upper and lower CI values of the regression estimates of the slope and then transforming them using Equation (1). Significance levels of differences between rates of decline of different alleles between different time periods, and when including vs. excluding post-treatment samples, were tested by *t*-tests. data from a previously published study in Malawi [3] were analysed in the same manner. However, in that study, the frequencies of resistant mutants were calculated as the proportion of all samples with both single and mixed genotypes that carried the resistant allele, either alone or with wild-type alleles. Thus estimates of population allele frequency of the mutants in that study are over-estimates.

## Results

Four hundred and six, 240 and 323 isolates were genotyped for *pfcrt-76*, *pfmdr1–86 *and *dhfr*, respectively, collected between 1993 and 2006 in Kilifi. Of these, 41, 28 and 58 carried mixed alleles and were not used in the analysis. Data on *pfcrt *genotypes from patients that had been treated with AQ (in 2006) were excluded because post-treatment patients harboured a significantly higher frequency of the *pfcrt-76 *mutant than pre-treatment patients (19/19 vs. 19/31, *P *= 0.002). There was also an indication that AL selected against the *pfcrt *mutant allele (frequencies of 6/8 pre-treatment vs. 2/7 post-treatment, P = 0.13). There were no significant effects of any other drugs used in the clinical trials on *pfmdr-86 *and *dhfr *allele frequencies. Nor did the inclusion of these post-treatment samples alter the slope of the regression lines in analyses of time trends in allele frequencies. Nevertheless, for the final analyses of time trends, Data from post-treatment samples were excluded. The total number of samples in these analyses was 322, 169 and 248 for *pfcrt*, *pfmdr *and *dhfr*, respectively, with medians of 29, 20 and 23 per year (Additional file 1).

### *Pfcrt-76 *and *pfmdr1–86*

**Figure 1:**
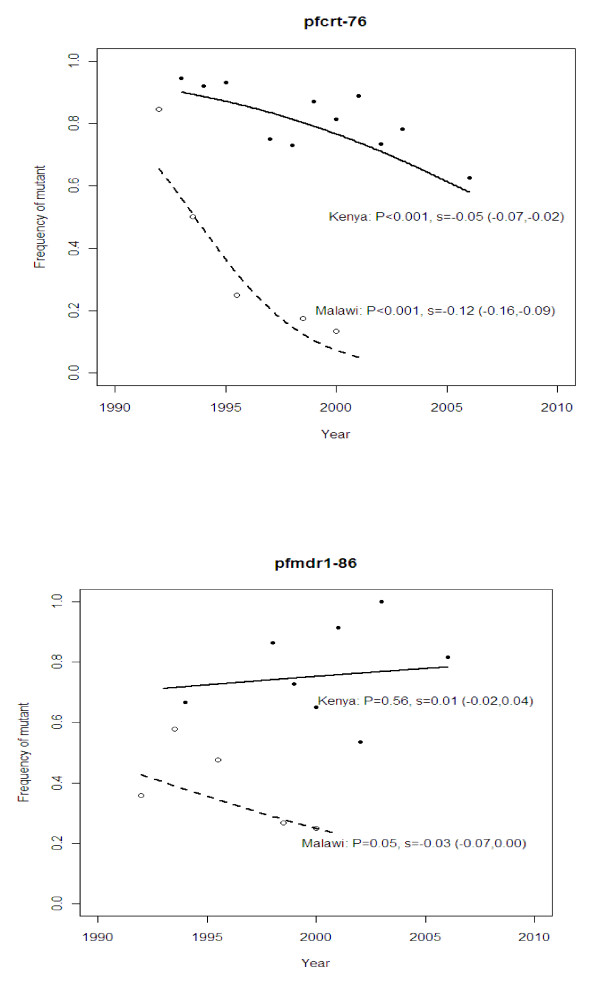
**Observed and fitted (by logistic regression) frequencies of *pfrt-76 *and *pfmdr1–86 *through time in Kilifi, Kenya and a study in Malawi **[3]. Observed (symbols) and predicted (lines) population frequencies through time of alleles *pfcrt-76 *(top panel) and *pfmdr1–86 *(bottom panel) involved in resistance to chloroquine in Kenya (solid line, closed symbols) and Malawi (dashed line, open symbols) before and after the official ban on the use of chloroquine (1999 and 1992, respectively). The *P*-value indicates whether the slope of the predicted line is significantly different from zero. The selection coefficient (*s*) of the resistant allele relative to the wild-type allele is shown with its 95% confidence intervals for each location.

In Kilifi, the frequency of the *pfcrt-76 *mutant significantly decreased (P < 0.001) from around 94% to 63% over a period of 13 years (Figure 1a). This decline was occurring before the official withdrawal of CQ in 1999 (P < 0.05). There was no detectable difference in the rate of decline before and after this time (*P *> 0.9 by test for an interaction between the slope and time period). The estimated fitness of this resistant mutant was 5% lower than that of the wild-type (*s *= -0.05). In Malawi, the rate of decline after CQ was withdrawn was much faster than in Kilifi (from 85% to 13% in 9 years). The estimated fitness of the resistant mutant in the Malawi study was 12% lower than that of the wild-type (*s *= -0.12): this was significantly greater in magnitude than that in the Kilifi study (*P *< 0.001, by Student's *t*-test of the logistic regression slopes).

*pfmdr-86 *did not decline significantly (P = 0.6) in the Kilifi population (Figure 1b), and had a corresponding small selection coefficient of 1%. On the other hand, in the Malawi population, the frequency of the *pfmdr1 *mutant allele decreased slightly (*P *= 0.05) with an estimated selection coefficient of -3%.

### *dhfr*

**Figure 2:**
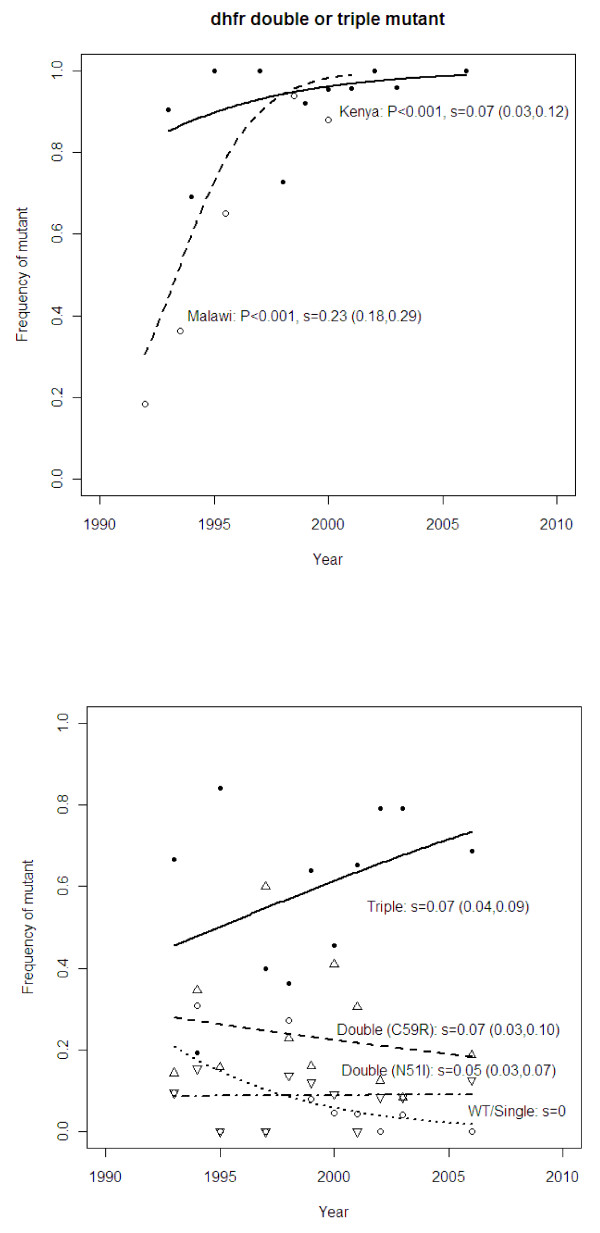
**Observed and predicted frequencies of *dhfr *mutations through time in Kilifi, Kenya and a study in Malawi **[3]. Observed (symbols) and predicted (lines) population frequencies through time of alleles at the *dhfr *locus determining resistance to pyrimethamine, one of the partner drugs in SP. This drug officially replaced CQ as the first line drug in 1990 and 1992 in Kenya and Malawi, respectively: however, in Kenya, it was used as a second line drug prior to the change from CQ. In the upper panel, the data are for the frequency of the double and triple mutant alleles combined whereas in the lower panel, the alleles are treated separately (see Methods). Selection coefficients are expressed relative to the wild-type/single mutant genotype.

In Kilifi, the frequency of the *dhfr *double and triple mutants combined was already high (around 80%) at the start of the study (five years before SP became the first-line treatment for uncomplicated malaria in Kenya). Nonetheless, this frequency increased significantly (*P *< 0.001) to above 95% during the study period (Figure 2a). Relative to the wild-type/single mutants, the triple and double mutants combined had an estimated selection coefficient of 7%. In contrast, the change in frequency in Malawi was faster (from 15% to above 80% within nine years) with a selection coefficient of 23% for the double and triple mutants combined. This was significantly higher than that for Kilifi (*P *< 0.001 by Student's *t*-test of the regression slopes). No isolate had a mutation at 164 codon of *dhfr*.

Analysis of the mutant alleles separately in the Kilifi data showed that the selection coefficients of the *dhfr *Asn51Ile-Cyst59Arg double mutants were 5% and 7%, respectively, and 8% for the triple mutant. The frequencies of the double mutants stayed approximately the same through time because, at the same time as they were gaining ground to the wild-type/single mutant, they were losing ground to the triple mutant (Figure 2b). The values of *s *found here span the range of estimates from other sites in Africa and South-East Asia [31]. The effect of including mixed infections on estimates of *s *was negligible. Thus the inability to detect alleles at low frequencies within a patient sample by the genotyping methods used here is unlikely to influence the results.

### Prediction of *pfcrt*, *pfmdr1 *and *dhfr *allele frequencies in the future

Based on the above estimates and Equation (1), it is predicted that, if the current drug pressure were to be maintained, Kilifi isolates will have largely reverted to CQ-sensitive phenotypes (frequency of the *pfcrt-76 *mutant less than 10%) by the year 2023, over 20 years after the official removal of the drug in Kenya. This contrasts with the case in Malawi where this level of sensitivity was reached within nine years following CQ withdrawal. In Kilifi, virtually no change is expected in *pfmdr1–86 *frequency while in Malawi by 2009, the frequency of *pfmdr1–86 *mutant is expected to fall below 10%.

### In vitro activity

**Figure 3:**
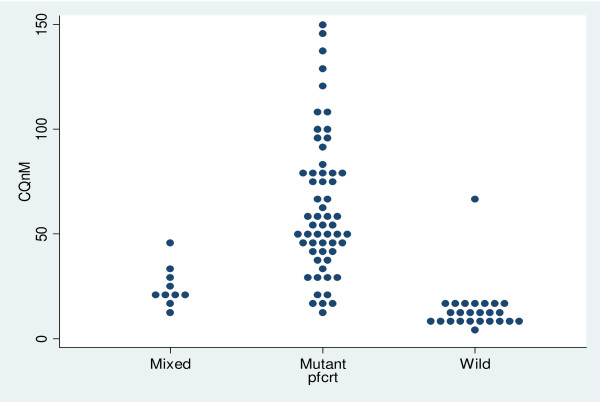
**Chloroquine *in vitro *activity and pfcrt-76 genotype**. Relationship between chloroquine inhibitory concentration that kills 50% of parasitaemia (IC_50_) as measured by 50% inhibition of [^3^H]hypoxanthine incorporation into parasite nucleic acid, and *pfcrt *genotype at codon 76 of isolates collected in 2006 in Kilifi. The dashed line indicates the cut-off level of 25 nM.

CQ IC_50 _against laboratory reference strains V1S, W2 (*pfcrt-76 *mutant) and 3D7 and M24 (wild type) were analysed and and these IC_50 _values are 158 ± 75, 94.15 ± 16, 6.5 ± 2.3, 15.6 ± 8.6 nM respectively. The results of field isolates are summarized in Figure 3. Depending on their *pfcrt-76 *genotypes, parasites can be classified into three distinct groups. The first group, the wild-type group, has IC_50 _values ranging between 1 and 25 nM, with an average of 13 ± 12 nM. The second group is composed of mixed genotype infections (wild-type and mutant), with a mean of 24 ± 10 nM; and the third group of *pfcrt-76 *mutants, which forms the majority of isolates, has a mean of 63 ± 90 nM (ranging from 5 to 150 nM). To distinguish CQ-sensitive and CQ-resistant isolates, a cut-off point of 100 nM is generally used [32,33]. However, using this cut-off point, most isolates would be classified as CQ-sensitive, yet they are *pfcrt-76 *mutant. Thus, the most accurate cut-off point to use in our setting would be 25 nM (Figure 3). The analysis of *pfmdr1–86 *genotype showed that wild-type isolates had lower CQ IC_50 _than mutant (57 ± 50 nM versus 68 nM+87 nM), but this difference was not statistically significant (p > 0.05).

## Discussion

The results show that CQ resistance in Kilifi was in decline since the early 1990s despite CQ only being officially withdrawn from Kenya in 1999 [5] when it was replaced by SP. In Kilifi District, this replacement was effective, largely owing to an educational programme for rural drug retailers during the change-over period in 1998 to 2001, which raised the proportion of drug users that purchased adequate doses of the first-line drug over-the-counter (OTC) from 8% to 64% [34]. Thus, following this successful training programme, CQ was largely discontinued in Kilifi, and replaced by a high usage of SP. In other sites in Kenya, however, CQ remained available at the community level as a drug for self-medication, even four years after its withdrawal [35]. The rate of change before and after the point of official withdrawal (2001) was not detectably different (P > 0.05).

These results suggest that widespread clinical failure of CQ prior to the ban drove people to use alternative drugs such as SP (which was then the second line of treatment) regardless of the prevailing official policy. This is supported by the high frequency of the *dhfr *double and triple mutants in the Kilifi population from the early 1990s [18] (Figure 2). Furthermore, prior to 2001, even though CQ was the most used anti-malarial, in most cases this drug was used at sub-therapeutic doses [34]. Thus it seems that declining usage and low efficacy due to high rates of clinical failure led to a decline in resistance well before its official discontinuation.

The fact that the frequency of the mutant allele nevertheless declined before the ban during continued use of the drug implies that there is a considerable cost of the *pfcrt *mutation to the parasite in the absence of drugs as even a small amount of drug pressure is sufficient to counteract quite high fitness costs [28]. That this is the case is evident from the 12% lower relative fitness estimated for Malawi after the drug was completely withdrawn. The apparently smaller relative fitness cost of 5% in Kilifi supports our view that the drug also remained in circulation despite the ban, although we consider alternative explanations below.

The frequency of the *pfmdr1 *mutant did not significantly decrease over time, and this could be explained by the marginal role *pfmdr1 *plays in CQ-resistance compared to *pfcrt *[36,37]. This is supported by the results of this study showing that the *pfmdr1 *mutants do not have higher CQ IC_50_s than wild type. However, in Malawi, over few years, the frequency of *pfmdr1 *mutants significantly decreased, and this may be due to a rapid decline of CQ-resistance. Thus in Kenya, this may be observed when the *pfcrt *wild type reaches a high frequency in the population.

It is well established that mutations that render an organism resistant to a drug are likely to be associated with a loss of fitness. As a result, organisms carrying these mutations will be outgrown by drug-sensitive organisms when the drug pressure is removed or reduced [38-42]. An increase in CQ *in vitro *activity and a decrease in prevalence of *pfcrt-76 *was also observed after the withdrawal of CQ in China [43,44]. Selection of CQ resistance was associated with a loss of fitness in murine malaria, and the same phenomenon has been reported for other anti-malarial drugs, such as pyrimethamine and atovaquone in *P. falciparum *[45-47]. In some instances, loss of fitness may be associated with the development of compensatory mechanisms, leading to persistence of the mutant parasite in the population despite the discontinuation of the drug. This feature may explain, at least in part, the persistence of *pfcrt-76 *mutant in parts of South-East Asia and South America [48-50], and in Kilifi as described here, despite CQ not having been used in these areas for many years.

The apparent difference between Kenya and Malawi in the rate of disappearance may also have been due to different usages rates of AQ, a close analogue to CQ. This and other studies have demonstrated that the use of AQ can select for the *pfcrt*-76 mutant [51]. AQ has been the second line of treatment in Kenya since SP was introduced in 1999 and has remained so until now. This drug has partially been used in several sites in Kenya [35], including Kilifi, even before the withdrawal of CQ [34].

CQ *in vitro *activity of Kenyan isolates collected in 2006 in this population were also tested. The data showed that the cut-off point of 100 nM [33] commonly used to distinguish CQ-resistant isolates from CQ-sensitive ones is not appropriate in our setting. We propose that 25 nM is a more suitable cut-off point to use in Kilifi, which is much lower than in other areas. The most likely explanation of this low cut-off point is the variability of the chemosensitivity assay in different settings due to the many factors that influence it [32,52]. For example, here we used the CQ-resistant strains V1S and W2 and the CQ-sensitive M24 and 3D7 as controls. The observation of previous reports show that CQ IC_50 _against these strains are 0.7 time lower to four times higher than IC_50 _values we obtained, and in general, our IC_50_s against the CQ-resistant strains V1S and W2 were at least 1.5 time lower [32,53-59]. Thus, whilst the *in vitro *chemosensitivity assay is a robust technique to discriminate between CQ-sensitive and CQ-resistant isolates within a setting, there is a need to define the cut-off point in every research setting [32,60].

## Conclusion

The current report demonstrates that CQ-resistance has been decreasing in Kilifi since its withdrawal in 1999, but that this decline was much slower than in Malawi. It will take 13 more years for the clinical efficacy of CQ to be restored in Kilifi. The most likely reason for this difference is that the change of drug policy from CQ to SP in Malawi was swift and effective, such that SP became the only available anti-malarial drug for treatment within one year of implementaiton. In Kenya, it appears that the ban was both too late and was ineffectively applied, so that it gave rise to the use of other drugs such as AQ, as well as continued use of CQ, thereby maintaining selection for CQ resistance. Thus, while the highly efficient implementation of drug policy in Malawi that permitted a faster reversal of CQ resistance also promoted more rapid selection for resistance against the replacement drug, SP, than in Kenya, the less rigorous enforcement of drug policy in Kenya has resulted in both the high maintenance of CQ resistance as well as high SP resistance. Thus the effectiveness of drug policy implementation can have important and far-reaching effects on the useful life of life-saving drugs. The newer policies of using drugs in combinations in order to prolong the resistance-free period is a good example of this [28,61].

## Abbreviations

CQ: Chloroquine; *dhfr*: dihydrofolate reductase; SP: sulphadoxine/pyrimethamine; Lapdap™: chlorproguanil/dapsone; Artekin^®^: piperaquine-dihydroartemisinin; AQ: amodiaquine; AL: artemether/lumefantrine; Lys: lysine; Thr: treonine; Asn: asparagine; Tyr: tyrosine; IC_50_: concentration required for 50% inhibition of [^3^H] hypoxanthine incorporation into parasite nucleic acid; WT: wild type; OTC: over-the-counter.

## Competing interests

The authors declare that they have no competing interests.

## Authors' contributions

LM carried out parasite adaptation, drug chemo-sensitivity assays and genetic analysis. EO contributed in the genotyping of pfcrt, pfmdr1 and dhfr genes. AA and SM contributed in the *in-vitro *culture and drug sensitivity assays. SW, GK, KM and AN designed the study and were involved in manuscript development. PS and SB contributed in generating isolates that were used for the assessment of CQ activity. MM carried out statistical analyses, and contributing in manuscript development.

## Supplementary Material

Additional file 1 **Numbers of genotypes carrying the mutant form (out of total number of genotypes) after excluding mixed infections and post-treatment samples**. Supplemental table. Click here for file

## References

[B1] Peters W (1971). Malaria. Chemoprophylaxis and chemotherapy. BMC.

[B2] Bloland PB, Ettling M (1999). Making malaria-treatment policy in the face of drug resistance. Ann Trop Med Parasitol.

[B3] Kublin JG, Cortese JF, Njunju EM, Mukadam RA, Wirima JJ, Kazembe PN, Djimde AA, Kouriba B, Taylor TE, Plowe CV, Contreras CE, Caraballo A (2003). Reemergence of chloroquine-sensitive *Plasmodium falciparum *malaria after cessation of chloroquine use in Malawi. J Infect Dis.

[B4] Laufer MK, Thesing PC, Eddington ND, Masonga R, Dzinjalamala FK, Takala SL, Taylor TE, Plowe CV (2006). Return of chloroquine antimalarial efficacy in Malawi. N Engl J Med.

[B5] Shretta R, Omumbo J, Rapuoda B, Snow RW (2000). Using evidence to change antimalarial drug policy in Kenya. Trop Med Int Health.

[B6] Eriksen J, Nsimba SE, Minzi OM, Sanga AJ, Petzold M, Gustafsson LL, Warsame MY, Tomson G (2005). Adoption of the new antimalarial drug policy in Tanzania–a cross-sectional study in the community. Trop Med Int Health.

[B7] Kamya MR, Bakyaita NN, Talisuna AO, Were WM, Staedke SG (2002). Increasing antimalarial drug resistance in Uganda and revision of the national drug policy. Trop Med Int Health.

[B8] Mita T, Kaneko A, Lum JK, Bwijo B, Takechi M, Zungu IL, Tsukahara T, Tanabe K, Kobayakawa T, Bjorkman A (2003). Recovery of chloroquine sensitivity and low prevalence of the *Plasmodium falciparum *chloroquine resistance transporter gene mutation K76T following the discontinuance of chloroquine use in Malawi. Am J Trop Med Hyg.

[B9] Takechi M, Matsuo M, Ziba C, MacHeso A, Butao D, Zungu IL, Chakanika I, Bustos MD (2001). Therapeutic efficacy of sulphadoxine/pyrimethamine and susceptibility *in vitro *of *P. falciparum *isolates to sulphadoxine-pyremethamine and other antimalarial drugs in Malawian children. Trop Med Int Health.

[B10] Gregson A, Plowe CV (2005). Mechanisms of resistance of malaria parasites to antifolates. Pharmacol Rev.

[B11] Sibley CH, Hyde JE, Sims PF, Plowe CV, Kublin JG, Mberu EK, Cowman AF, Winstanley PA, Watkins WM, Nzila AM (2001). Pyrimethamine-sulfadoxine resistance in *Plasmodium falciparum*: what next?. Trends Parasitol.

[B12] Amukoye E, Winstanley PA, Watkins WM, Snow RW, Hatcher J, Mosobo M, Ngumbao E, Lowe B, Ton M, Minyiri G, Marsh K (1997). Chlorproguanil-dapsone: effective treatment for uncomplicated falciparum malaria. Antimicrob Agents Chemother.

[B13] Makanga M, Premji Z, Falade C, Karbwang J, Mueller EA, Andriano K, Hunt P, De Palacios PI (2006). Efficacy and safety of the six-dose regimen of artemether-lumefantrine in pediatrics with uncomplicated *Plasmodium falciparum *malaria: a pooled analysis of individual patient data. Am J Trop Med Hyg.

[B14] Sulo J, Chimpeni P, Hatcher J, Kublin JG, Plowe CV, Molyneux ME, Marsh K, Taylor TE, Watkins WM, Winstanley PA (2002). Chlorproguanil-dapsone versus sulfadoxine-pyrimethamine for sequential episodes of uncomplicated falciparum malaria in Kenya and Malawi: a randomised clinical trial. Lancet.

[B15] Winstanley P, Watkins W, Muhia D, Szwandt S, Amukoye E, Marsh K (1997). Chlorproguanil/dapsone for uncomplicated *Plasmodium falciparum *malaria in young children: pharmacokinetics and therapeutic range. Trans R Soc Trop Med Hyg.

[B16] Sasi P, Abdirahman A, Mwai L, Muriithi S, Straimer J, Schieck L, Rippert A, Bashraheil M, Salim A, Peshu J, Awuondo K, Lowe B, Pirmohamed M, Winstanley P, Ward S, Nzila A, Borrmann S (2009). In vivo and in vitro efficacy of amodiaquine against *Plasmodium falciparum *in an area of continued use of 4-aminoquinolines in East Africa. J Infect Dis.

[B17] Sixsmith DG, Watkins WM, Chulay JD, Spencer HC (1984). In vitro antimalarial activity of tetrahydrofolate dehydrogenase inhibitors. Am J Trop Med Hyg.

[B18] Nzila AM, Mberu EK, Sulo J, Dayo H, Winstanley PA, Sibley CH, Watkins WM (2000). Towards an understanding of the mechanism of pyrimethamine-sulfadoxine resistance in *Plasmodium falciparum*: genotyping of dihydrofolate reductase and dihydropteroate synthase of Kenyan parasites. Antimicrob Agents Chemother.

[B19] Nzila AM, Nduati E, Mberu EK, Hopkins Sibley C, Monks SA, Winstanley PA, Watkins WM (2000). Molecular evidence of greater selective pressure for drug resistance exerted by the long-acting antifolate Pyrimethamine/Sulfadoxine compared with the shorter-acting chlorproguanil/dapsone on Kenyan *Plasmodium falciparum*. J Infect Dis.

[B20] Nzila A, Ochong E, Nduati E, Gilbert K, Winstanley P, Ward S, Marsh K (2005). Why has the dihydrofolate reductase 164 mutation not consistently been found in Africa yet?. Trans R Soc Trop Med Hyg.

[B21] Ochong E, Nzila A, Kimani S, Kokwaro G, Mutabingwa T, Watkins W, Marsh K (2003). Molecular monitoring of the Leu-164 mutation of dihydrofolate reductase in a highly sulfadoxine/pyrimethamine-resistant area in Africa. Malar J.

[B22] Hamel MJ, Poe A, Bloland P, McCollum A, Zhou Z, Shi YP, Ouma P, Otieno K, Vulule J, Escalante A, Udhayakumar V, Slutsker L (2008). Dihydrofolate reductase I164L mutations in *Plasmodium falciparum *isolates: clinical outcome of 14 Kenyan adults infected with parasites harbouring the I164L mutation. Trans R Soc Trop Med Hyg.

[B23] Juliano JJ, Trottman P, Mwapasa V, Meshnick SR (2008). Detection of the dihydrofolate reductase-164L mutation in *Plasmodium falciparum *infections from Malawi by heteroduplex tracking assay. Am J Trop Med Hyg.

[B24] Lynch C, Pearce R, Pota H, Cox J, Abeku TA, Rwakimari J, Naidoo I, Tibenderana J, Roper C (2008). Emergence of a dhfr mutation conferring high-level drug resistance in *Plasmodium falciparum *populations from southwest Uganda. J Infect Dis.

[B25] Nair S, Williams JT, Brockman A, Paiphun L, Mayxay M, Newton PN, Guthmann JP, Smithuis FM, Hien TT, White NJ, Nosten F, Anderson TJ (2003). A selective sweep driven by pyrimethamine treatment in southeast asian malaria parasites. Mol Biol Evol.

[B26] Roper C, Pearce R, Bredenkamp B, Gumede J, Drakeley C, Mosha F, Chandramohan D, Sharp B (2003). Antifolate antimalarial resistance in southeast Africa: a population-based analysis. Lancet.

[B27] Molineaux L, Gramiccia G (1980). The Garki Project: Research on the Epidemiology and Control of Malaria in the Sudan Savanna of West Africa.

[B28] Mackinnon MJ, Hastings IM (1998). The evolution of multiple drug resistance in malaria parasites. Trans R Soc Trop Med Hyg.

[B29] Haldane JBS (1924). A mathematical theory of natural and artificial selection. Trans Cambridge Philos Soc.

[B30] Østergård H (1987). Estimating relative fitness in asexually reproducing plant pathogen populations. Theor Appl Genet.

[B31] Anderson TJ (2004). Mapping drug resistance genes in *Plasmodium falciparum *by genome-wide association. Curr Drug Targets Infect Disord.

[B32] Briolant S, Parola P, Fusai T, Madamet-Torrentino M, Baret E, Mosnier J, Delmont JP, Parzy D, Minodier P, Rogier C, Pradines B (2007). Influence of oxygen on asexual blood cycle and susceptibility of *Plasmodium falciparum *to chloroquine: requirement of a standardized in vitro assay. Malar J.

[B33] Le Bras J, Ringwald P (1990). *Plasmodium falciparum *chemoresistance. The situation in Africa in 1989. Med Trop (Mars).

[B34] Marsh VM, Mutemi WM, Willetts A, Bayah K, Were S, Ross A, Marsh K (2004). Improving malaria home treatment by training drug retailers in rural Kenya. Trop Med Int Health.

[B35] Amin AA, Snow RW (2005). Brands, costs and registration status of antimalarial drugs in the Kenyan retail sector. Malar J.

[B36] Duraisingh MT, Cowman AF (2005). Contribution of the pfmdr1 gene to antimalarial drug-resistance. Acta Trop.

[B37] Ekland EH, Fidock DA (2007). Advances in understanding the genetic basis of antimalarial drug resistance. Curr Opin Microbiol.

[B38] Austin DJ, Anderson RM (1999). Studies of antibiotic resistance within the patient, hospitals and the community using simple mathematical models. Philos Trans R Soc Lond B Biol Sci.

[B39] Bouma JE, Lenski RE (1988). Evolution of a bacteria/plasmid association. Nature.

[B40] Lenski RE (1998). Bacterial evolution and the cost of antibiotic resistance. Int Microbiol.

[B41] Levy SB (1994). Balancing the drug-resistance equation. Trends Microbiol.

[B42] Walliker D (2005). The hitchhiker's guide to malaria parasite genes. Trends Parasitol.

[B43] Laufer MK, Plowe CV (2004). Withdrawing antimalarial drugs: impact on parasite resistance and implications for malaria treatment policies. Drug Resist Updat.

[B44] Wang X, Mu J, Li G, Chen P, Guo X, Fu L, Chen L, Su X, Wellems TE (2005). Decreased prevalence of the *Plasmodium falciparum *chloroquine resistance transporter 76T marker associated with cessation of chloroquine use against P. falciparum malaria in Hainan, People's Republic of China. Am J Trop Med Hyg.

[B45] Peters JM, Chen N, Gatton M, Korsinczky M, Fowler EV, Manzetti S, Saul A, Cheng Q (2002). Mutations in cytochrome b resulting in atovaquone resistance are associated with loss of fitness in *Plasmodium falciparum*. Antimicrob Agents Chemother.

[B46] Rosario VE, Hall R, Walliker D, Beale GH (1978). Persistence of drug-resistant malaria parasites. Lancet.

[B47] Shinondo CJ, Lanners HN, Lowrie RC, Wiser MF (1994). Effect of pyrimethamine resistance on sporogony in a *Plasmodium berghei*/Anopheles stephensi model. Exp Parasitol.

[B48] Cortese JF, Caraballo A, Contreras CE, Plowe CV (2002). Origin and dissemination of *Plasmodium falciparum *drug-resistance mutations in South America. J Infect Dis.

[B49] Vieira PP, das Gracas Alecrim M, da Silva LH, Gonzalez-Jimenez I, Zalis MG (2001). Analysis of the PfCRT K76T mutation in *Plasmodium falciparum *isolates from the Amazon region of Brazil. J Infect Dis.

[B50] Zalis MG, Pang L, Silveira MS, Milhous WK, Wirth DF (1998). Characterization of *Plasmodium falciparum *isolated from the Amazon region of Brazil: evidence for quinine resistance. Am J Trop Med Hyg.

[B51] Ochong EO, Broek IV van den, Keus K, Nzila A (2003). Short report: association between chloroquine and amodiaquine resistance and allelic variation in the *Plasmodium falciparum *multiple drug resistance 1 gene and the chloroquine resistance transporter gene in isolates from the upper Nile in southern Sudan. Am J Trop Med Hyg.

[B52] Bacon DJ, Jambou R, Fandeur T, Le Bras J, Wongsrichanalai C, Fukuda MM, Ringwald P, Sibley CH, Kyle DE (2007). World Antimalarial Resistance Network (WARN) II: in vitro antimalarial drug susceptibility. Malar J.

[B53] Foote SJ, Kyle DE, Martin RK, Oduola AM, Forsyth K, Kemp DJ, Cowman AF (1990). Several alleles of the multidrug-resistance gene are closely linked to chloroquine resistance in *Plasmodium falciparum*. Nature.

[B54] Lakshmanan V, Bray PG, Verdier-Pinard D, Johnson DJ, Horrocks P, Muhle RA, Alakpa GE, Hughes RH, Ward SA, Krogstad DJ, Sidhu AB, Fidock DA (2005). A critical role for PfCRT K76T in *Plasmodium falciparum *verapamil-reversible chloroquine resistance. Embo J.

[B55] Mu J, Ferdig MT, Feng X, Joy DA, Duan J, Furuya T, Subramanian G, Aravind L, Cooper RA, Wootton JC, Xiong M, Su XZ (2003). Multiple transporters associated with malaria parasite responses to chloroquine and quinine. Mol Microbiol.

[B56] Oduola AM, Milhous WK, Weatherly NF, Bowdre JH, Desjardins RE (1988). *Plasmodium falciparum*: induction of resistance to mefloquine in cloned strains by continuous drug exposure in vitro. Exp Parasitol.

[B57] Oduola AM, Weatherly NF, Bowdre JH, Desjardins RE (1988). *Plasmodium falciparum*: cloning by single-erythrocyte micromanipulation and heterogeneity in vitro. Exp Parasitol.

[B58] Su X, Kirkman LA, Fujioka H, Wellems TE (1997). Complex polymorphisms in an approximately 330 kDa protein are linked to chloroquine-resistant *P. falciparum *in Southeast Asia and Africa. Cell.

[B59] Suwanarusk R, Russell B, Chavchich M, Chalfein F, Kenangalem E, Kosaisavee V, Prasetyorini B, Piera KA, Barends M, Brockman A, Lek-Uthai U, Anstey NM, Tjitra E, Nosten F, Cheng Q, Price RN (2007). Chloroquine resistant *Plasmodium vivax*: In vitro characterisation and association with molecular polymorphisms. PLoS ONE.

[B60] Durand R, Jafari S, Vauzelle J, Delabre JF, Jesic Z, Le Bras J (2001). Analysis of pfcrt point mutations and chloroquine susceptibility in isolates of *Plasmodium falciparum*. Mol Biochem Parasitol.

[B61] White NJ (2004). Antimalarial drug resistance. J Clin Invest.

