# Supplementary material for: Chloroquine resistance before and after its withdrawal in Kenya
Source: Malar J. 2009 May 18;8:106. doi: 10.1186/1475-2875-8-106 (PMC2694831; doi:10.1186/1475-2875-8-106)
Supplement: Additional file 1 — Numbers of genotypes carrying the mutant form (out of total number of genotypes) after excluding mixed infections and post-treatment samples. Supplemental table. [file 1475-2875-8-106-S1.doc]

**Supplementary information**

Table S1. Numbers of genotypes carrying the mutant form (out of total number of genotypes) after excluding mixed infections and post-treatment samples.

| **Year** | ***pfcrt-76*** | ***pfmdr1-86*** | ***dhfr-108**** | ***dhfr-108-51*** | ***dhfr-108-59*** | ***Dhfr108-51-59*** |
| --- | --- | --- | --- | --- | --- | --- |
| **1993** | 17/18 | - | 2/21 | 3/21 | 2/21 | 14/21 |
| **1994** | 35/38 | 10/15 | 8/26 | 9/26 | 4/26 | 5/26 |
| **1995** | 27/29 | - | 0/19 | 3/19 | 0/19 | 16/19 |
| **1997** | 12/16 | - | 0/10 | 6/10 | 0/10 | 4/10 |
| **1998** | 19/26 | 19/22 | 6/22 | 5/22 | 3/22 | 8/22 |
| **1999** | 27/31 | 16/22 | 2/25 | 4/25 | 3/25 | 16/25 |
| **2000** | 22/27 | 13/20 | 1/22 | 9/22 | 2/22 | 12/22 |
| **2001** | 32/36 | 21/23 | 1/23 | 7/23 | 0/23 | 8/23 |
| **2002** | 22/30 | 15/28 | 0/24 | 3/24 | 2/24 | 5/24 |
| **2003** | 18/23 | 1/1 | 1/24 | 2/24 | 2/24 | 19/24 |
| **2006** | 30/48 | 31/38 | 0/32 | 6/32 | 4/32 | 22/32 |
|  |  |  |  |  |  |  |

* They also included wild type dhfr
